# Copaiba oil minimizes inflammation and promotes parenchyma re-epithelization in acute allergic asthma model induced by ovalbumin in BALB/c mice

**DOI:** 10.3389/fphar.2024.1356598

**Published:** 2024-04-11

**Authors:** Ludmila de Souza Caputo, Carolina de Lima Alves, Inês Martins Laranjeira, Diana Fonseca-Rodrigues, Ademar Alves da Silva Filho, Alberto Carlos Pires Dias, Filipa Pinto-Ribeiro, Olavo dos Santos Pereira Junior, Ana Claudia Chagas de Paula, Akinori Cardozo Nagato, José Otávio do Amaral Corrêa

**Affiliations:** ^1^ Department of Pharmaceutical Sciences, Federal University of Juiz de Fora, Juiz de Fora, Brazil; ^2^ Life and Health Sciences Research Institute, ICVS, School of Medicine, Campus of Gualtar, University of Minho, Braga, Portugal; ^3^ ICVS/3B‟s - PT Government Associate Laboratory, Braga, Portugal; ^4^ Centre for the Research and Technology of Agro-Environmental and Biological Sciences, CITAB, University of Trás-os-Montes e Alto Douro, Vila Real, Portugal; ^5^ Centre of Molecular and Environmental Biology, CBMA, University of Minho, Campus de Gualtar, Braga, Portugal; ^6^ Department of Physiology, Federal University of Juiz de Fora, UFJF, Juiz de Fora, Brazil

**Keywords:** asthma, lung inflammation, airway remodeling, natural products, Copaifera langsdorffii Desf, anti-inflammatory agent, re-epithelialization

## Abstract

**Introduction:** Asthma is a condition of airflow limitation, common throughout the world, with high mortality rates, especially as it still faces some obstacles in its management. As it constitutes a public health challenge, this study aimed to investigate the effect of copaiba oil (e.g., *Copaifera langsdorffii*), as a treatment resource, at doses of 50 and 100 mg/kg on certain mediators of acute lung inflammation (IL-33, GATA3, FOXP3, STAT3, and TBET) and early mechanisms of lung remodeling (degradation of elastic fiber tissues, collagen deposition, and goblet cell hyperplasia).

**Methods:** Using an ovalbumin-induced acute allergic asthma model in BALB/c mice, we analyzed the inflammatory mediators through immunohistochemistry and the mechanisms of lung remodeling through histopathology, employing orcein, Masson’s trichrome, and periodic acid-Schiff staining.

**Results:** Copaiba oil treatment (CO) reduced IL-33 and increased FOXP3 by stimulating the FOXP3/GATA3 and FOXP3/STAT3 pathways. Additionally, it upregulated TBET, suggesting an additional role in controlling GATA3 activity. In the respiratory epithelium, CO decreased the fragmentation of elastic fibers while increasing the deposition of collagen fibers, favoring epithelial restructuring. Simultaneously, CO reduced goblet cell hyperplasia.

**Discussion:** Although additional research is warranted, the demonstrated anti-inflammatory and re-epithelializing action makes CO a viable option in exploring new treatments for acute allergic asthma.

## 1 Introduction

Asthma is a chronic inflammatory condition of the airways characterized by variable airflow limitation and caused by external environmental stimuli associated with epigenetic factors. More than 262 million people are affected by this debilitating disease, whose symptoms include shortness of breath, chest tightness, wheezing, and cough (Global Asthma Network, 2022). In Brazil, the incidence varies between regions around 19.8%–24.9% (Brazil, 2022). During crises, clinical outcomes could range from high hospitalizations to death (approximately 1,000 per day) (Global Asthma Network, 2022). Moreover, around 10%–15% of patients experience a permanent loss of lung function during their entire life, even when treated with bronchodilators and steroids (Aegerter and Lambrecht, 2023).

Disease classification into phenotypes makes individual treatment choices more assertive. Allergic asthma is the most easily recognized phenotype based on demographic, clinical, and pathophysiological characteristics. However, more research is necessary to prove the exact relationship between pathological outcomes and particular clinical patterns in each group of patients (Global Asthma Network, 2022; Aegerter and Lambrecht, 2023).

Asthma endotypes distinguish the pathophysiologic mechanisms at a cellular and molecular level (Kuruvilla et al., 2019) and are defined by the main subset of T cells involved in the disease. In susceptible individuals, allergic ones, the recurrent contact of irritating factors with the respiratory epithelium causes damage. Consequently, alarmins, such as IL-33, are released to promote eosinophilic recruitment and the activation of mast cells and CD4^+^ T cells (T helper cells, Th) in the mucosa, as well as the expression of high-affinity IgE receptors on inflammatory and resident cells (Akar-Ghibril et al., 2020).

Classically, Th1, Th2, Th17, and Treg specific immune responses are described in allergic lung inflammation (Yang et al., 2019; Luo et al., 2022; Jiang et al., 2023). The Th2 high asthma endotype is commonly associated with reactions induced by allergens, microbes, pollutants, and fungi that damage the epithelial barrier and stimulate goblet cells to produce mucus (Lourenço et al., 2022). Due to the predominance of Th2 lymphocytes, this profile stands out for its high eosinophilic recruitment. The polarization of Th1 and Th17 cells with neutrophil recruitment and lower Th2 response (paradigm of the Th cell response) are associated with the severity of the immune response. In contrast, Treg cells appear to counterregulate the intensity of these responses (Zhu et al., 2020).

IL-33 orchestrates the responses of Th2 cells and regulates the production of IL-17A and IL-31 cytokines (Gauvreau et al., 2020). The activation of the transcription factor GATA-binding protein 3 (GATA3) and its sustained expression, in the presence of a signal of transducer and activator of transcription (STAT) 5, promote the differentiation of naïve T cells into Th2 cells, resulting in the translation and secretion of standard cytokines: IL-4, IL-5, IL-9, and IL-13 (Komlósi et al., 2022). The presence of IL-33 appears to enhance signaling mediated by GATA3 and STAT5, maintaining the predominance of Th2 at the site of inflammation (Peine et al., 2016). On the other hand, if the transcription factors orphan retinoic acid receptors (RORγt)/STAT3 and T-box expressed in T cells (TBET) are activated, T naïve differentiates, respectively, into Th17 (IL-17, IL-22) and Th1 (Interferon gamma, IFN-γ) cells, exacerbating the inflammatory response, especially with intense neutrophil recruitment (Gauvreau et al., 2023). In the presence of Forkhead box protein P3 (FOXP3), T naïve cells are differentiated into Treg cells and promote paracrine effects at the inflammatory site through IL-10 secretion (Griesenauer and Paczesny, 2017).

Corticosteroids and β2-agonists are references in asthma treatment, but some individuals respond refractorily (Pavord et al., 2018; Gubernatorova et al., 2019), and monoclonal antibodies are a phenotype-specific therapy that still presents a high cost (Solé et al., 2019). Besides that, even today, available treatments, especially for exacerbated cases, provide symptom relief, not a cure (Global Asthma Network, 2022; World Health Organization, 2023), and remain associated with adverse events, especially in the resistant population (Pavord et al., 2018). Side effects with long-term high doses of inhaled corticosteroids include, for example, cataracts, glaucoma, adrenal suppression, growth retardation, etc. Those for oral use are associated with an increased risk of diabetes, obesity, reflux, hypertension, depression, anxiety, and thromboembolism, among others (Global Initiative for Asthma, 2022).

The diversity of therapy responses, especially on the part of individuals who have severity asthma, the difficulty in accessing treatment, the incidence of adverse effects associated with recently used therapies, and low adherence to treatment regimen are some of the obstacles related to the management of asthma, at its different levels, and which has contributed to the high mortality rate, making its management a global challenge (Global Asthma Network, 2022).

Copaiba oil (CO) (e.g., *Copaifera lansgdorfii*) is a natural oil with anti-hemorrhoidal, purgative, anti-carcinogenic, antimicrobial, anesthetic, and anti-inflammatory properties (Cardinelli et al., 2023), traditionally used to treat diseases and inflammations of the skin (Lee, J. et al., 2023). Furthermore, it is popularly adopted in respiratory syndrome treatment (Leandro et al., 2012). Due to its anti-inflammatory activity, CO indicates that it has the potential to treat asthma with the advantage of presenting fewer side effects compared to corticosteroids (Menezes et al., 2022), in addition to having a low cost compared to other traditional therapies (Teixeira et al., 2017).

CO is mainly composed of sesquiterpenes and diterpenes in proportions that may vary according to the copaiba species and also due to the region, time, plant part, and the chosen extraction method (Cardinelli et al., 2023; Frazão et al., 2023). In *Copaifera lagsdorffii*, for example, α-copaene, α-selinene, α-humulene (α-hum), β-elemene, β-caryophyllene (BCP), and caryophyllene oxide have been documented in the literature as constituents of oils found in the majority of Brazilian regions where this species thrives (Santos et al., 2022). Oils of *C. officinalis* have in their composition α-Copaene and oils of *C. multijuga* contain BCP, α-hum, and α-bergamotene as main components. Because of its abundance, BCP is the chemical marker of copaiba oil in some species. Both BCP and α-hum exhibit anti-inflammatory effects (Frazão et al., 2023) and have been explored as treatment alternatives in certain asthma experimental models, demonstrating the ability to regulate immune aspects (Rogerio et al., 2009; Pathak et al., 2021; Wei et al., 2021). Other components, such as α-copaene and α-bergamotene present antioxidant properties (Frazão et al., 2023), useful to treat asthma oxidative stress. However, despite the effects described for some isolated compounds, most of the biological properties are attributed to the intact oil only, where the substances may act synergistically (Cardinelli et al., 2023).

In an animal model of traumatic ulcers, Alvarenga et al. (2020) demonstrated the potential of CO to accelerate re-epithelialization and minimize the inflammatory process. According to the authors, this effect is probably related to the synergistic effect between BCP and α-hum present in the oil, which impacts the proliferative phase of collagen organization and increases the secretion of IL-8, related to angiogenesis.

In an experimental model of allergic asthma, our research group was able to demonstrate that the treatment with CO reduced the infiltrate of eosinophils and neutrophils, also reducing the dosage of nitrite and the concentration of IL-4, IL-5, IgE, IL-17, TNF-α and IFN-γ related to Th2, Th17 and Th1 responses (Caputo et al., 2020). In a complementary manner, the present study aimed to evaluate if CO modulates the expression of IL-33 and to investigate whether this response pattern was related to the deviation of the immune response towards Th1, Th2, and Th17 cells. We also intend to verify how it can act on some mechanisms of lung remodeling.

## 2 Materials and methods

### 2.1 Copaiba oil and reagents

The CO came from Brazil, obtained from the trunk of copaiba trees belonging to *Copaifera langsdorffii.* It was acquired from Pharmanostra (actual InfinityPharma) (São Paulo, SP, Brazil), lot 16E09-B027-009523. The chemical characterization of this tested CO was performed previously by GC-MS and GC-FID (Caputo et al., 2020).

OVA was from Sigma Aldrich^®^ (São Paulo, SP, Brazil), Ketamine (10%) from Syntec^®^ (São Paulo, SP, Brazil), and Xylazine (2%) from Ceva^®^ (São Paulo, SP, Brazil). The Star Trek Universal HRP Detection System kit (STUHRP700H, L10) was from Biocare Medical (Pacheco, CA, USA). The primary antibodies anti-GATA3, anti-STAT3, anti-TBET, and anti-FOXP3 were obtained from Santa Cruz Biotechnology (Dallas, Texas, USA), and the anti-IL-33 and the goat IgG hrp-conjugated antibody were from VWR International (Carnaxide, Portugal).

### 2.2 Mice

The investigation worked with twenty-five female BALB/c mice (8 weeks old) obtained from the Center for Biology of Reproduction at the Federal University of Juiz de Fora (UFJF, Juiz de Fora, MG, Brazil). Mice stayed in polypropylene cages under controlled temperature (∼22°C), fluorescent and cyclical lighting (12 h of light/12 h of darkness - lights on at 6 a.m.), receiving water and palletized food *ad libitum*. The University Ethical Committee on the Use of Animals (UFJF/CEUA) approved all care and experimental procedures, protocol nº. 015/2016, being the experimentation also in compliance with the Brazilian Guide for the Production, Maintenance, or Use of Animals in Teaching or Scientific Research Activities.

### 2.3 Asthma induction and treatment

**FIGURE 1:**
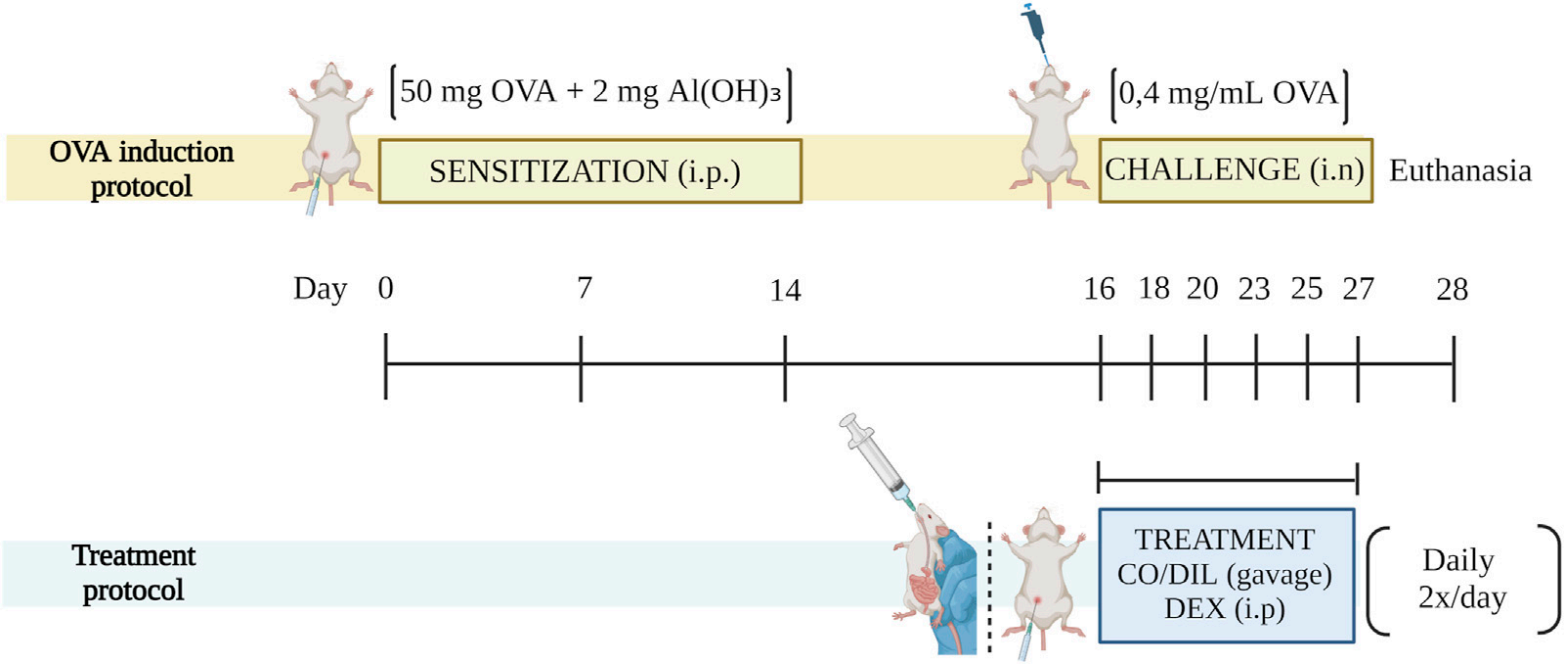
Scheme of the experimental model and treatment protocol. DIL: diluent. SOURCE: Prepared by the author. Created with BioRender.com.

OVA induction and treatment scheme was carried out with five animal groups (5 animals/each) as described in our previous study (Caputo et al., 2020) (Figure 1). In four of them, sensitization was performed through intraperitoneal (i.p.) injection of 50 mg of OVA +2 mg of aluminum hydroxide (Al(OH)_3_) dissolved in 200 uL of buffered saline solution (PBS), pH 7, 4, followed by intranasal (i.n.) challenge with 0.4 mg/mL of OVA dissolved in PBS. Separately, the fifth group received saline solution instead of OVA in the steps described previously, being considered the CONTROL group.

The treatments began parallel with the challenge, initially administered 2 h after the challenge, remaining daily, every 12 h. The time interval was determined based on intranasal or gastric antigen administration studies that indicate the allergens reach a plasma peak between 1 and 2 h after intranasal administration (Passalacqua et al., 2005). Around 14% of OVA is absorbed through the respiratory mucosa (Hens et al., 2007) while the gastrointestinal tract absorbs most of the allergen, and OVA serum concentrations decrease significantly around 120 min (Matsubara et al., 2008).

The experimental groups received the following names based on the method of inducing inflammation and the treatment adopted: I. CONTROL, II. OVA, III. OVA + DEX (2 mg/kg), IV. OVA + CO 50 mg/kg and V. OVA + CO 100 mg/kg. The CONTROL and OVA groups received diluent (2% Tween 80) by gavage in place of active ingredients. Dexamethasone was administered i.p. by the superior absorption of this route compared to the enteral one. CO, as the object of study, was administered by oral gavage due to the increased comfort of this route.

Euthanasia was carried out through the administration of a lethal dose of anesthesia (xylazine/ketamine). The animals then underwent lobectomy and the right lobes were sent for histopathological processing, which consists of longitudinal sectioning, fixation in 4% formalin for 24 h, dehydration, diaphanization, and paraffin inclusion. Tissue sections of 3–5 μm thickness were arranged in slides and directed for subsequent tests.

### 2.4 Immunohistochemical analysis (IHC)

The IHC analysis of IL-33, GATA3, FOXP3, STAT3, and TBET expression followed standard laboratory protocols. Silanized slides containing lung tissue were dewaxed and used for testing. Assays with anti-GATA3, anti-STAT3, anti-TBET, and anti-FOXP3 antibodies, diluted 1:100, were performed using the Star Trek Universal HRP Detection System kit (Biocare Medical, CA, USA) in which the secondary antibody is biotinylated and forms a complex with HRP-streptavidin. The assay with anti-IL-33 antibody (1:200) followed the indirect method using the secondary antibody conjugated to HRP. All samples were stained by the chromogenic substrate diaminobenzidine (DAB) and counterstained with Hematoxylin. We also performed negative controls without the primary antibodies.

After completing the tests, we analyzed the slides by light microscopy (400x) using 15 photomicrographs of the parenchyma per section collected by the AxioCam CHF 5 capture system—Carl Zeiss Microscopy GmbH, 2011 (Zeiss, Berlin, Germany) by measuring the density of the parenchyma area stained with DAB (mm^2^) per field, corrected by the parenchyma area (Vvpar) and subtracting the area occupied by the air space (Aar) from the total image area (Aimage) for the adjustment, so that Vvpar = Aimage–Aar, as described by Nagato et al. (2015).

### 2.5 Histopathological analyzes

The orcein staining was conducted to verify the degradation of elastic fibers resulting from the inflammatory process. The Masson Trichrome (TM) technique helped to complement the analysis of epithelial damage staining the collagen in the alveolar epithelium, which made it possible to evaluate the collagen deposition between the experimental groups. Both techniques were analyzed by light microscopy (400x) using 15 photomicrographs of the parenchyma per section (Zeiss, Berlin, Germany) and measuring the density of the stained area followed by area adjusting (Vvpar = Aimage–Aar).

Periodic acid-Schiff (PAS) counter-colored with HE was executed to detect goblet cell hyperplasia. Goblet cell number was quantified per 100 epithelial cells at randomly selected microscopy fields (400x) in 6 photomicrographs of bronchiolar regions per lung section, captured under light microscopy (Zeiss, Berlin, Germany).

### 2.6 Statistical analysis

The data were evaluated for normality using the D'Agostino–Pearson and Shapiro-Wilk tests. To compare the experimental groups, we employed one-way ANOVA with Tukey’s test for parametric data and the Kruskal–Wallis test with Dunn’s *post hoc* test for non-parametric data. We set the significance level at 5% (*p* < 0.05) and conducted the data analysis using GraphPad Prism version 8.0.1 for Windows (GraphPad Software, San Diego, CA), expressing the results as the arithmetic mean and standard error of the mean (S.E.M).

## 3 Results

### 3.1 CO reduces IL-33 levels

**FIGURE 2:**
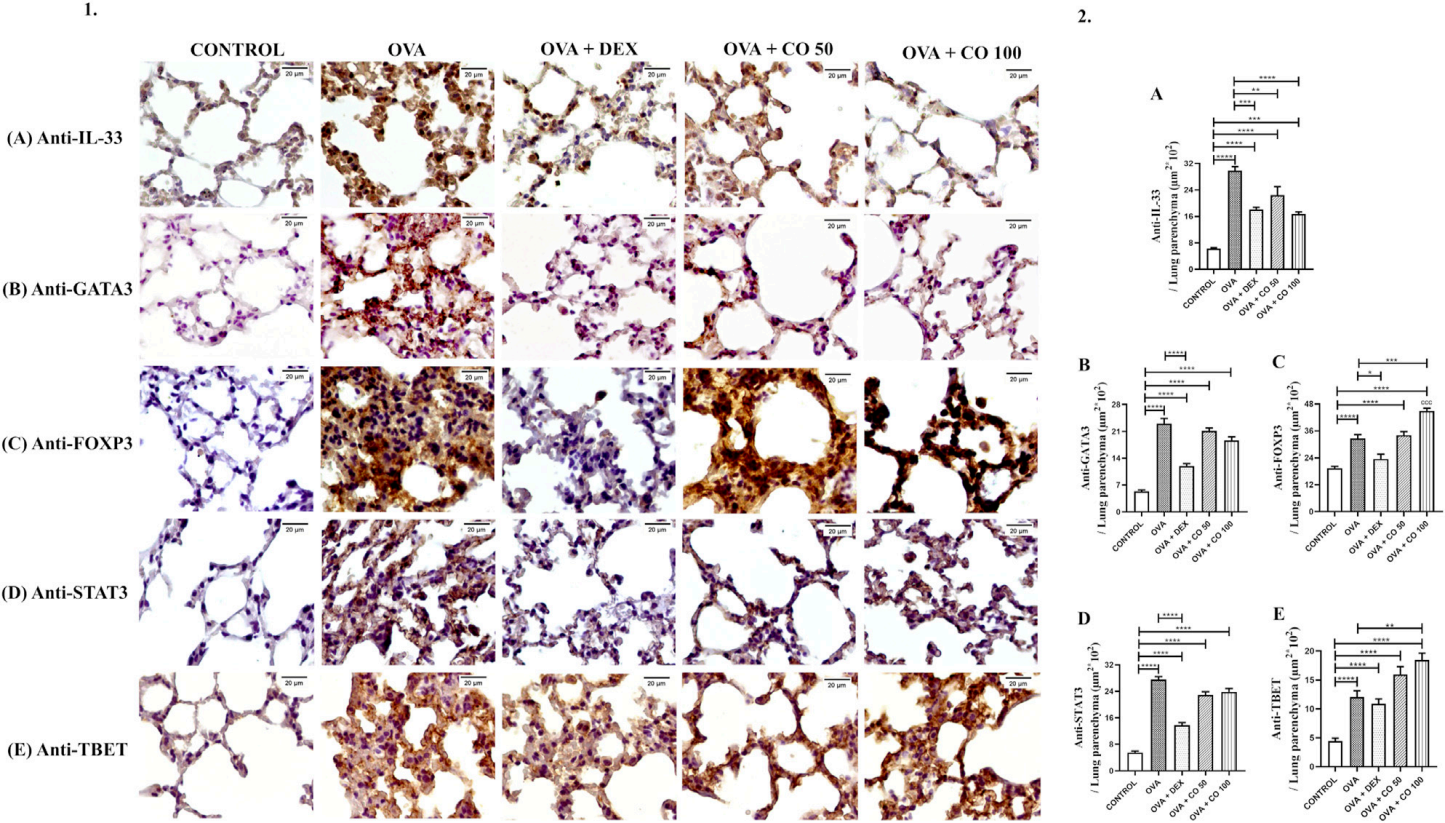
(1) Representative IHCs photomicrographs; (2) Density area of each antibody stain with DAB (mm2), per total parenchyma area. **(A)** Anti-IL-33, **(B)** anti-GATA-3, **(C)** anti-FOXP3 **(D)** anti-STAT3 and **(E)** anti-TBET. c: Statistical differences between OVA + CO 50 and OVA + CO 100. SOURCE: Prepared by the author.

Compared to the CONTROL group, OVA significantly increased the levels of IL-33 (*p* < 0.05), a cytokine related to the immediate reaction to epithelial damage and the Th2 response domain. Treatment with DEX and CO maintained lower levels of IL-33 compared to the latter group (*p* < 0.05). Incidentally, we did not observe significant differences between the DEX and CO groups (*p* > 0.05) (Figure 2A).

### 3.2 CO upregulates FOXP3 and TBET expression

The OVA group showed an increase in FOXP3 levels compared to the CONTROL group (*p* < 0.05), as an attempt by the animal’s immune system to regulate inflammatory mechanisms. In another direction, DEX treatment maintained FOXP3 at a similar level to the CONTROL group (*p* > 0.05). In contrast to DEX, CO upregulated FOXP3, and CO treatment at 100 mg/kg increased FOXP3 to levels even higher than those found in the OVA group (*p* < 0.05) (Figure 2C).

Like FOXP3, TBET levels increased in the OVA group in contrast to the CONTROL one (*p* < 0.05). Although the DEX treatment presents lower TBET levels than OVA, these were insignificant (*p* > 0.05). On the other hand, CO treatment at 100 mg/kg upregulated TBET levels compared to the OVA group (*p* < 0.05) (Figure 2E).

### 3.3 CO did not alter the levels of GATA3 and FOXP3, maintaining them at elevated levels

We observed a significant increase in GATA3 levels in the OVA compared to the CONTROL group (*p* < 0.05), which confirms the induction of the proposed experimental model, which presents a Th2 response profile (GATA3). As expected, DEX treatment maintained lower GATA3 levels compared to diluent-treated inflamed animals (*p* < 0.05). However, CO treatments did not significantly change GATA3 levels compared to the OVA group (*p* > 0.05) (Figure 2B).

In the same way, STAT3 levels increased in mice induced with inflammation (OVA group) in comparison to the CONTROL group (*p* < 0.05), which indicates the contribution of Th17 response in the model. DEX treatment maintained lower levels of STAT3 compared to the OVA group (*p* < 0.05), while CO did not significantly change levels compared to the same group (*p* > 0.05) (Figure 2D).

### 3.4 CO promotes re-epithelialization of the small airways

We evaluated three aspects related to epithelial damage and airway remodeling to investigate the scope of CO treatment on remodeling characteristics, specifically, the degradation of elastic fibers, collagen deposition, and goblet cell hyperplasia.

**FIGURE 3:**
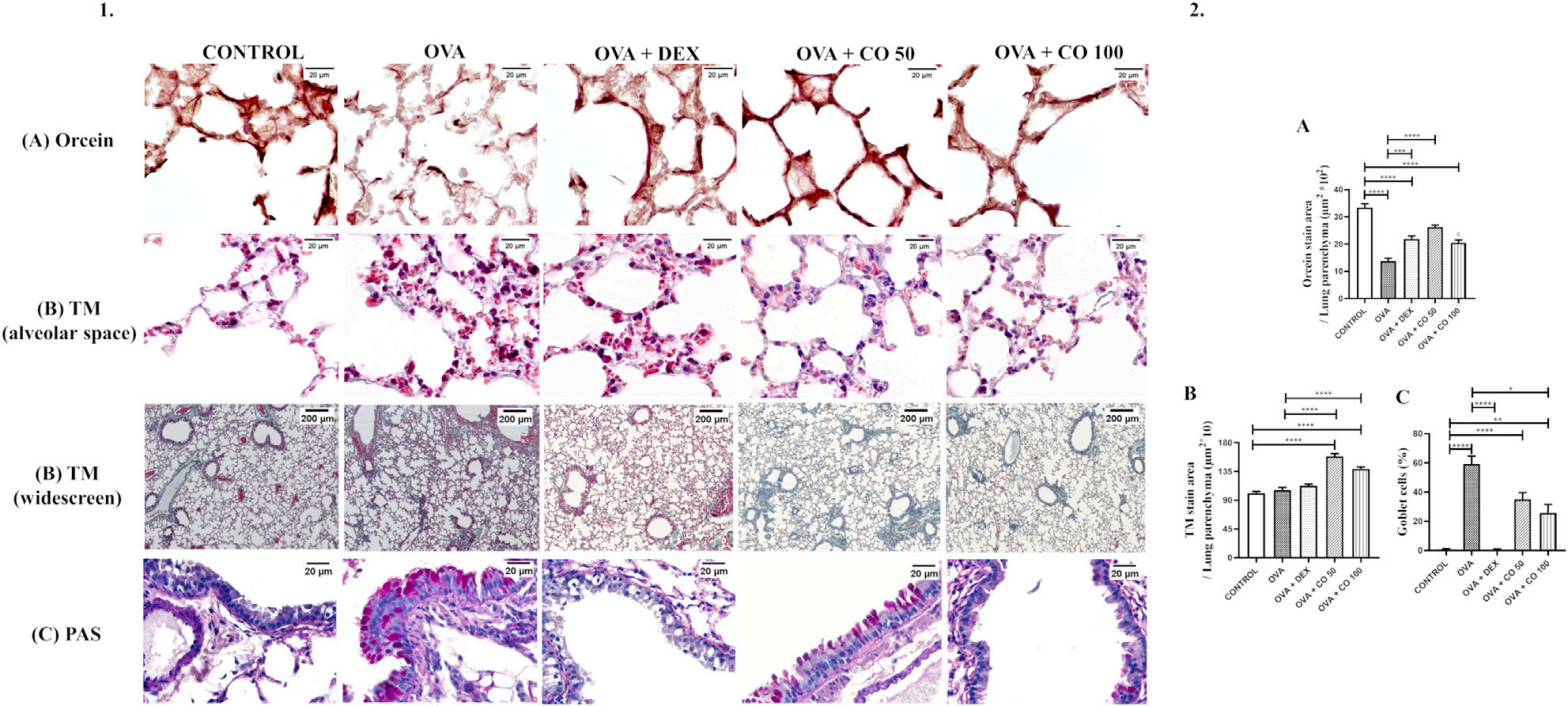
(1) Representative photomicrographs of the remodeling aspects analyzed in the lung parenchyma. (2) Density area of tissue stained by **(A)** Orcein, **(B)** TM per parenchyma area and **(C)** PAS. Percentage of goblet cells per experimental group. c: Statistical differences between OVA + CO 50 and OVA + CO 100. SOURCE: Prepared by the author.

In the lungs of animals induced with inflammation by OVA, degradation of elastic fibers was observed. It is represented in a graph by the total area stained by orcein, in which it is possible to verify a significant difference between the area corresponding to the CONTROL and the OVA group (*p* < 0.05). Although the treatment groups differed from the CONTROL one (*p* < 0.05), it was possible to verify that DEX and CO 50 mg/kg reduced the degradation observed, presenting a more positive stain area than the OVA group (*p* < 0.05) (Figure 3A).

Collagen deposition did not show significant differences between the CONTROL, OVA, and DEX groups (*p* > 0.05). At the same time, CO increased the amount of collagen in both concentrations (*p* < 0.05), compared to CONTROL and OVA groups) (Figure 3B).

Mice induced with inflammation also presented goblet cell hyperplasia compared to the CONTROL group (*p* < 0.05). DEX and CO at 100 mg/kg reduced the percentage of goblet cells compared to OVA animals (*p* < 0.05) (Figure 3C). Figure 6 summarizes all CO targets investigated.

## 4 Discussion

**FIGURE 4:**
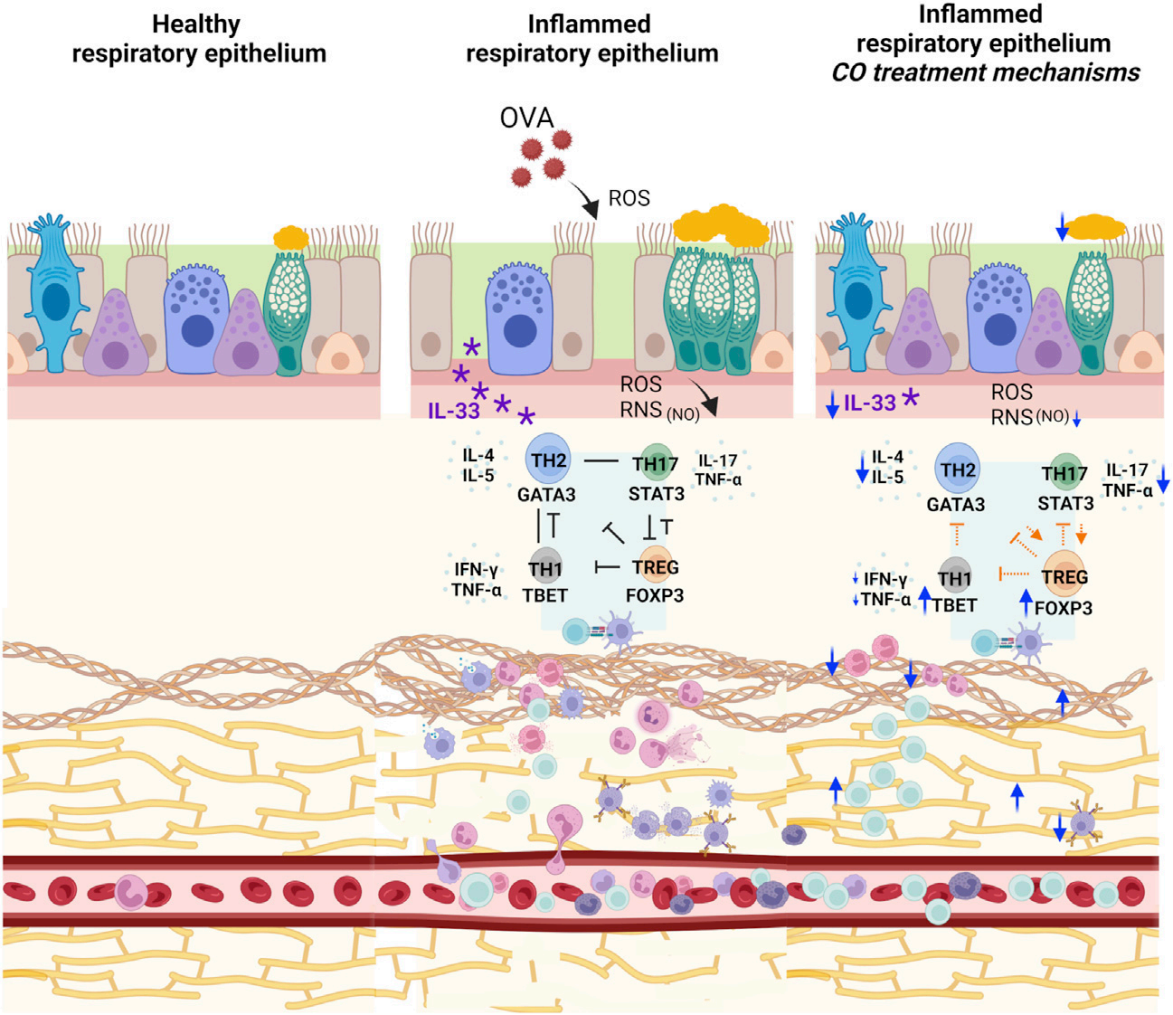
Representation of CO action mechanisms in the experimental model. Administration of CO concomitantly with OVA challenges reduced IL-33, goblet cell hyperplasia, and degeneration of elastic fibers with a simultaneous increase in collagen fibers. The treatment increased FOXP3 directly and indirectly by maintaining elevated GATA3 and STAT3 compared to the CONTROL group. Furthermore, CO promoted an increase in TBET, balancing the exacerbation of the inflammatory response promoted by GATA3. In addition, in our previous findings, CO reduced the number of eosinophils and neutrophils and increased the T lymphocyte count. We also verified a reduction in the concentration of IL-4, IL-5, IL-17, TNF-α, and IFN-γ and a reduction in nitrite dosage (Caputo et al., 2020). SOURCE: Prepared by the author. Created with BioRender.com.

The interaction between the immune system and tissue remodeling in asthma necessitates the proposition of a comprehensive treatment regimen or a broad-spectrum anti-inflammatory approach for enhanced disease control (Maltby et al., 2017; Banno et al., 2020). Preliminary research from our group in an asthma model induced with OVA in BALB/c mice showed that CO treatment regulates eosinophils, neutrophils, and the inflammatory markers IL-4, IL-5, IgE, IL-17, TNF-α, and IFN-γ (Figure 4). It also decreases nitrite dosage and restores tissue structure, including the hyperplasia and hypertrophy of the smooth muscle, vascular congestion, and the thickening of the alveolar wall. Our Copaiba oil, previously analyzed by CG-MS and CG-FID, is constituted of 16 compounds, with the major ones being BCP (62.8%), α-hum (9.1%), α-copaene (5.1%), and bergamotene (4.4%) (Caputo et al., 2020). All components exhibit synergistic or additive effects related to the action targets of this product.

In our recent findings, we noted an increase in IL-33 levels of OVA compared to the CONTROL group (*p* < 0.05). This elevation in IL-33 levels demonstrates a positive correlation with the severity of symptoms, as highlighted in the study by Gauvreau et al. (2023). This nuclear cytokine is constitutively expressed in mice, particularly in the alveolar epithelium, with a notable presence in type II alveolar cells. Additionally, it is found in certain microvascular beds and stromal cells (Cayrol and Girard, 2022).

Allergens that affect the airways are capable of cleaving IL-33 through proteases. The same also occurs in inflammatory cells. Cleavage, in turn, makes IL-33 more active than its intact form. However, in allergens that do not have proteolytic activity, such as OVA (Lewkowich et al., 2011), uncleaved IL-33 acts less pronounced, which is why it only stimulates the type 2 endotype when present in large quantities. Subsequently, in chronic phases of inflammation, proteolytic enzymes secreted by neutrophils and mast cells appear to contribute to the cleavage of IL-33 (Cayrol and Girard, 2022) and an increase in its activity (Huang et al., 2022).

Our previous findings (Caputo et al., 2020) showed an increase in the number of neutrophils, possibly related to IL-33 cleavage, in bronchoalveolar lavage fluid (BALF) and the total count of inflammatory cells in the parenchyma. We also verified that CO treatment reduced the population of these inflammatory cells, as mentioned. Complementarily, in the current data, CO reduced IL-33 levels compared to the OVA group (*p* < 0.05) (Figure 4).

Based on the literature, we hypothesize that the action of CO may be associated with the modulation of the mitogen-activated protein kinase (MAPK) signaling pathway. In a study on carbon-black-induced lung inflammation, macrophage-induced MAPK activation increased IL-33 expression by regulating the transcription of pro-inflammatory cytokines (Cheng et al., 2019). Additionally, previous research in liver cancer cells demonstrated that CO could stimulate the MAPK pathway, leading to increased phosphorylation of mitogen-activated ERK kinase 1/2 (MEK1/2) and, consequently, extracellular signal-regulated kinase1/2 (ERK 1/2) activation (Urasaki and Le, 2019). Moreover, another investigation revealed that the BCP compound could downregulate MAPK by inhibiting the phosphorylation of transforming growth factor-β-activated kinase 1 (TAK1), a component of the MAPK signal, and by increasing the expression of MAP kinase phosphatase 1 (MKP1) in bone marrow-derived macrophages stimulated with LPS (Zhang et al., 2021).

Regarding transcription factors, GATA3 is expressed in hematopoietic and myeloid cells and is essential for T cell development, DCs, mast cells, eosinophils, erythrocytes, and platelets (Garn and Renz, 2017). As previously described (Lee B. et al., 2018; Aslani et al., 2022), GATA3 was upregulated in the OVA compared to the CONTROL group (*p* < 0.05) Although positive GATA3 mRNA signals are commonly localized in infiltrating inflammatory cells (Nakamura et al., 1999), type II alveolar cells also express GATA3 and are involved in the production of Th2 cytokines (Keil et al., 2020), as we have seen.

Even though CO has reduced IL-33, GATA3 labeling reduction between the OVA and CO groups did not occur significantly (*p* > 0.05). Nevertheless, a significant reduction in the concentration of IL-4, IL-5, and IgE in the BALF and lung macerate of BALB/c mice treated with CO at 100 mg/kg verified in our previous findings (Caputo et al., 2020), reinforces that CO exerts some modulation on Th2 signaling. As a result, we hypothesize that another mechanism of action for CO is the modulation of the GATA3/FOXP3 pathway (Figure 4). GATA3 also plays a crucial role in the function of Treg cells, which, as the name suggests, regulate the inflammatory response (Chen T. et al., 2018).

The phosphorylation and recruitment of GATA3 play a crucial role in the activation of Treg lineage transcription factor FOXP3 and the Il1rl1 locus. This process enhances the production of FOXP3, promoting differentiation in favor of Treg cells (Peine et al., 2016; Griesenauer and Paczesny, 2017). Additionally, the regulation of the CCR8 GATA3 receptor facilitates the migration of Tregs to the inflammatory site, thereby promoting asthma control (Chen W. et al., 2022). We have confirmed that OVA-exposed animals exhibited a significant increase in positive staining for FOXP3 compared to the CONTROL group (*p* < 0.05). According to the literature, both interstitial and bronchial epithelial cells express FOXP3 (Wang et al., 2022).

In the asthma model induced by OVA in BALB/c mice, Zhang et al. (2014) demonstrated an increase in both FOXP3 mRNA and protein levels in the lung tissue of asthmatic mice compared to the control group. According to the authors, the presence of the allergen in the airways leads to an augmentation of Treg, promoting immunological tolerance. In the present investigation, it seems that OVA induction has resulted in the generation of Tregs with insufficient capacity to suppress the Th2 response. This deficiency could be related, to some extent, to the upregulation of IL-33. Further studies are required to confirm our speculations.

Regarding CO effects, we demonstrated that CO at 100 mg/kg positively regulated FOXP3 compared to the OVA group (*p* < 0.05) (Figure 4). Furthermore, the observed increase in IL-10 with CO treatment (Amorim et al., 2017; Kian et al., 2018) supports our hypothesis and the results found. The obtained results align with existing literature data. Wei et al. (2021) investigated the impact of BCP in a model of neutrophilic asthma induced in BALB/c mice. They found that this compound activated the cannabinoid receptor type 2 (CNR2), leading to an increase in the expression of STAT5. STAT5, in turn, affected Treg cell differentiation in the lung, likely by promoting sustained GATA3 expression. In the study, differentiation of Treg cells by CNR2 modified the Treg/Th17 balance in favor of Tregs (*in vitro* tests) and increased IL-10 levels. Together, these mechanisms alleviated neutrophilic asthma. Another investigation in the experimental model of autoimmune encephalomyelitis induced in C57BL/6 mice by Askari and Shafiee-Nick (2019) demonstrated that treatment with BCP led to an increase in FOXP3 compared to the inflamed group that did not receive this compound.

However, the developmental pathways of Tregs and Th17 cells are reciprocally regulated by tissue growth factor (TGF-β), with stimulation of FOPX3 expression or RORγt, Th17 transcription factor. So, the natural mechanism of FOXP3 inhibition consists of inhibiting the differentiation of CD4^+^ T cells into Tregs by DCs matured by IL-33 and inducing the polarization of stable Tregs towards IL-17-producing cells through IL-6/IL-1β signaling (Park et al., 2020). The IL-6 increases RORγt transcription through signal transducer and activator of transcription 3 (STAT3), while TGF-β regulates Th17 differentiation. Phosphorylated by IL-6, STAT3 negatively regulates FOXP3 by binding to a silencing element within the FOXP3 locus, limiting its expression. This situation affects the stability of these cells, leading to the loss of their suppressive capacity and, consequently, to the induction of RORγt with the transformation of inducible Tregs into Th17 cells (Lan et al., 2020; Park et al., 2020; Paroli et al., 2022).

The contribution of Th17 cells in the model is associated with the severity of the inflammatory condition with an increase in the number of neutrophils (Luo et al., 2022). Among the cytokines produced by Th17 cells, IL-17A itself has the potential to activate STAT3 phosphorylation in naïve Th cells, helping to promote the IL-6/STAT3 pathway (Nikolskii et al., 2021).

In our findings, we observed an upregulation of STAT3 in OVA compared to the CONTROL group (*p* < 0.05), as verified by Xue et al. (2021). STAT3 was visualized in epithelial tissue and type II alveolar cells, as previously described by Yan et al. (2002). DEX treatment reduced STAT3 significantly (*p* < 0.05), but we did not find significant differences in the CO compared to the OVA group (*p* > 0.05). Based on this context, we hypothesized that CO may also act through the STAT3/FOXP3 pathway by modulating cytokines related to STAT3 phosphorylation, maintaining STAT3 levels, and promoting anti-inflammatory effects through FOXP3 (Figure 4).

In general, the suppressor of cytokine signaling 3 (SOCS3), regulates the JAK2/STAT3 pathway (Nikolskii et al., 2021) resulting in a rapid decline in the phosphorylation and presence of STAT3 in the nucleus (Cevey et al., 2019). However, STAT3 phosphorylation can also occur via JAK1/IL-10 and stimulate the suppression of pro-inflammatory effects. For example, STAT3 suppresses signaling from Toll like receptors (TLRs) in macrophages. STAT3 also participates in the induction of regulatory B cells (Bregs) secreting IL-10 and IL-35, in addition to initiating the expression of maturation factors and production of IgG antibodies, when activated in B cells, by follicular helper T cells (Tfh) (Nikolskii et al., 2021). The SOCS3 does not block the activation of STAT3 by IL-10, inducing the sustainment of STAT3 activation (Cevey et al., 2019).

Also, in our previous findings, CO (100 mg/kg) reduced IL-17 levels in BALF and lung macerate, demonstrating some inhibition of the Th17 response (Caputo et al., 2020). In a complementary way, Urasaki and Le (2019) treated human liver cancer cells with CO, showing that the treatment upregulated the JAK/STAT signaling pathway by increasing the phosphorylation of STAT3. Furthermore, Wei et al. (2021) found that BCP, trough CNR2 activation stimulate the AP-1 pathway and cooperates with STAT3 in promoting FOXP3 expression. Given that BCP is the main compound in CO, we believe that this same effect can also occur with the administration of CO. Still, more detailed studies are needed to support the hypothesis raised in this current investigation.

Like Th17 cells, Th1-mediated response also contributes to the severity of the inflammatory condition, abetted by IL-33 with the release of IFN-γ and TNF (Pinto et al., 2018). TBET is the main factor that regulate the magnitude of IFN-γ production, the critical signal of Th1 cell differentiation (Lu D. et al., 2020; Pang et al., 2021). In the current data, we observed an alveolar increase in TBET in the OVA compared to the CONTROL group (*p* < 0.05). According to Lu et al. (2011), in IHCs, this expression is mainly in the alveolar, peribronchial, and perivascular tissue of the animals induced to lung inflammation. It led us to believe that Th1 could have contributed to the neutrophil increase verified in our previous investigation in the OVA group. Nevertheless, this requires further clarification as IFN-γ levels remained statistically non-significant between the CONTROL and OVA group in BALF and lung macerate samples (Caputo et al., 2020).

Before, we observed that CO at 100 mg/kg could act in Th1 response mechanisms because it reduced the IFN-γ levels in the lung macerate compared to the OVA group (Caputo et al., 2020). Another investigation by our research group also showed that CO reduced IFN-γ in C57Bl/6 mice splenocytes induced by Experimental Autoimmune Encephalomyelitis (EAE) (Dias et al., 2014). Complementarily, in the EAE model with C57Bl/6 mice, BCP reduced the IFN-γ levels in the treated group compared with the EAE group (Fontes et al., 2017). In addition, a third study with the same model showed that BCP decreased TBET (*p* < 0.05) in the medulla and cerebellum of treated animals (Mesquita et al., 2019). Differently, in our present study, TBET was upregulated in the group treated with CO at 100 mg/kg compared to the OVA group (*p* < 0.05). Given this, we think this behavior could be an attempt to repress Th2 cell activation since Th1 can exercise an antagonistic role to confine eosinophilic inflammation (Zhu et al., 2020). Therefore, we assume that CO may be helping to maintain the balance between TBET/GATA3 (Figure 4).

The remodeling process may occur in parallel to inflammation and tends to persist independently of it (Saglani and Lloyd, 2015; Markus et al., 2017). In summary, in airway epithelial remodeling, an imbalance occurs between profibrotic and proteolytic factors in response to stress from successive allergen exposures, as an attempt to restore homeostasis, and such excessive changes, in turn, culminate in pronounced bronchoconstriction (Banno et al., 2020; Hough et al., 2020). Extracellular matrix (ECM) changes encompass the connective tissue composed of collagen and elastin fibers, mechanically connected via microfibrils/proteoglycans, and responsible for alleviating the primary stress of respiration, promoting structural integrity and elasticity (Oliveira et al., 2019; Mariano et al., 2023).

Granule proteins released during inflammatory cell degranulation and also proteolytic enzymes such as matrix proteins (MMPs) whose expression increases in stressed tissues can degenerate elastin (Yombo et al., 2023). The reduction of elasticity compromises expiratory elastic recoil which results in loss of patency of airway walls and gas trapping, increasing lung compliance and contributing to airway obstruction (Ingram et al., 2016; Zhou et al., 2019; Yombo et al., 2023). In this investigation, the OVA group presented damaged elastic fibers, while the CONTROL group presented intact fibers (*p* < 0.05). In parallel, in our previous study (Caputo et al., 2020), the OVA group showed an increase in eosinophils, neutrophils, and monocytes in the BALF, in addition to causing an increase in the dosage of nitrite in both BALF and lung macerate. All of which may have contributed to the process of degeneration (Zhou et al., 2019; Tehrani et al., 2021; Lin and Li, 2023; Thiam et al., 2023).

According to Rønnow et al. (2022) in the imbalance between MMPs and their inhibitors, an excessive level of MMPs can also lead to type I collagen degradation, one of the most abundant collagens in the healthy lung, crucial for providing mechanical stability and structure to the alveolar wall and septa. In agreement, another study found that primary airway fibroblasts from asthmatics, after 24 h of inflammatory stimuli (*in vitro*), show inefficient fibrillar collagen formation with fibers arranged in a disorganized manner. This occurrence was related to IL-1 signaling in the first hours of exposure to epithelial stress but was turned off later (Osei et al., 2020).

The separation of elastin from collagen by disruption can harm tissue integrity by weakening the collagen load-bearing threshold (Mariano et al., 2023). But, at the same time, in the repair process collagen fibers tend to accumulate and strengthen vulnerable areas, produced by fibroblasts and airway smooth muscle cells (Hough et al., 2020) which promotes tissue stiffening and decreases lung compliance (Mariano et al., 2023). At this time, the airway wall stiffening appears to be beneficial, helping with structural integrity and counterbalancing the increased constricting force of the thicker smooth muscle, which limit lumen occlusion (Setlakwe et al., 2014; Maghsoudi-Ganjeh et al., 2021), considering that smooth muscle cells lose direct cell–cell connections and form focal adhesions with collagen fibers in the underlying ECM to generate higher forces. Event that, in turn, reduces the ability of collagenase enzymes to effectively degrade collagen (Jamieson et al., 2021).

Despite in the current investigation, we did not observe any differences in collagen between the OVA group and the control group (*p* > 0.05), in the photomicrographs of the airways stained with TM, collagen appears to be more fragmented in the OVA than in the other groups. Similar to our research, Sakoda et al. (2016) induced BALB/c males to acute lung inflammation by OVA and also found no differences between parenchymal collagen content between the OVA and their control group.

We considered the possibility that collagen deposition might not have initiated within the period during which we conducted our analysis, 24 h after the last challenge with OVA. This consideration aligns with the findings of Savin et al. (2022), who demonstrated a significant increase in collagen content in the lung parenchyma and airways during the subacute phase in BALB/c mice induced with inflammation by OVA. These changes were observed 4 weeks after the last challenge with OVA, emphasizing that collagen deposition may occur in a delayed fashion. Reinforcing our hypothesis, Kenyon et al. (2003) only observed a significant increase in collagen in the airways of BALB/c from the fourth week of exposure to OVA, with a dose-response relationship throughout the induction time.

Regarding treatment effects in the connective tissue, CO at 50 mg/kg and DEX improved the integrity of elastic fibers compared to the OVA group (*p* < 0.05). CO, in truth, did not present statistical differences to the CONTROL group (*p* > 0.05). In addition, at both doses, CO increased collagen deposition compared to the other experimental groups (*p* < 0.05). Similarly, in a cutaneous wound healing model with Wistar rats, CO increased the number of elastic fibers and collagen compared to the group treated with saline solution. Moreover, the oil showed the potential to convert type III collagen (young) into type I collagen (mature), contributing to improved mechanical resistance and epithelial traction strength (Martini et al., 2016). Likewise, Biondo-Simões et al. (2019), who also evaluated the healing process in Wistar rats, found an increase in type I collagen in the group treated with CO compared to the group treated with saline solution.

Since we did not observe an increase in collagen deposition in the OVA group, we hypothesize that CO acts as an accelerator of the tissue remodeling process, simultaneously improving the elastic fibers’ integrity and increasing collagen deposition to stabilize the injured tissue and minimize airway occlusion (Figure 4). Even because, in a chronic model of asthma induced by OVA, IL-33 was able to stimulate fibroblasts to secrete type I collagen (Zhang et al., 2019).

In the dermis, CO stimulates the proliferation of fibroblasts and collagen (Palheta et al., 2017). It also promotes the synthesis of better-organized collagen fibers (Masson-Meyers et al., 2013). The organization of these fibers is important because it influences the direction of leukocyte migration (Mostaço-Guidolin et al., 2019). In collagen matrices, mechanical signals organize cellular arrangement in the ECM, facilitating cell alignment and cell-matrix clustering. As the main structural component of the ECM, collagen helps guide cell adhesion and tissue growth, playing a significant role in mediating cellular responses to injury (Liu et al., 2021). Thus, an action of CO on collagen would even provide better control over inflammatory cellular interaction.

The remodeling of elastic and collagen fibers, mainly in the walls of the airways, is also associated with increased mucus secretion and goblet cell hyperplasia (Ito et al., 2019). So, the induced model led to goblet cell hyperplasia (*p* < 0.05). Besides mucus secretion, goblet cells can produce some Th1, Th2, and Th17 cytokines and chemokines, as well as growth factors, contributing to immune hyperresponsiveness (Tanabe and Rubin, 2016). They act as primary sensors of environmental threats to the epithelial surface, and mucus interacts directly with DCs (Ma et al., 2017).

Treatment with CO at 100 mg/kg and DEX reduced the number of goblet cells compared to the OVA group (*p* < 0.05) (Figure 4). While there are no previous reports in the literature on the action of CO on goblet cells, there is evidence of the reparative effect of CO on gastric damage, where it increased gastric mucus to optimize protection against aggressive factors (Paiva et al., 1998). Some studies demonstrate that part of this activity appears to be exerted by the α-hum, present in CO (Yeo et al., 2021), and also by BCP, which inhibited mucus production in NCI-H292 cells (Lee H. J., 2017). Studies on gastric ulcers have indicated that mucins, present in mucus, are among the main protective components of the gastric mucosa, including MUC5AC. These mucins play a crucial role in protecting the gastric mucosa from offensive extrinsic factors (Yeo et al., 2021; Lee, D. et al., 2023). Similarly, in bronchial asthma, MUC5AC is produced extensively to protect the epithelium. However, its excessive synthesis can contribute to the occlusion of the airways, necessitating its suppression (Iwashita et al., 2022).

To conclude, we demonstrated that CO improved the inflammatory condition by reducing IL-33 and increasing FOXP3. We believe that CO also modulated GATA3/FOXP3 and STAT3/FOXP3 pathways to sustain FOXP3 anti-inflammatory response. Additionally, CO increased TBET levels acting in the Th2/Th1 balance, reducing type 2 exacerbated response. In the remodeling aspects, CO reduced the degradation of elastic fibers and increased the deposition of ECM collagen, improving the re-epitelization process and inflammatory cell migration from the tissue to the alveolar lumen. Furthermore, CO reduced the epithelial cells’ transition into mucus-producing goblet cells, helping to restore the airway’s lumen. Given all the data presented, we infer that CO contributed to restoring epithelial homeostasis in a non-dose-dependent manner, being a promising alternative to asthma treatment studies. However, certain crucial aspects, including lung mechanics and other remodeling markers like MMPs, as well as distinctions between collagen types, still require evaluation in the future to enhance our understanding of the conclusions drawn.

## Data Availability

The original contributions presented in the study are included in the article/Supplementary Material, further inquiries can be directed to the corresponding author.

## Abbreviations

BCP, β-caryophyllene; CO, Copaiba oil; DEX, dexamethasone; α-hum, α-humulene, UFJF, Federal University of Juiz de Fora.
